# Evaluating a novel automatic feeding device for hay provision in horses: behavioral outcomes in group and individual housing

**DOI:** 10.3389/fvets.2026.1891331

**Published:** 2026-10-06

**Authors:** Federica Raspa, Martina Favaro, Livio Penazzi, Chiara Vasile, Clara Bordin, Damiano Cavallini, Andrea Dorothea Ellis, Patricia Harris, Emanuela Valle

**Affiliations:** 1 Department of Veterinary Sciences, University of Turin, Grugliasco, Italy; 2 Department of Veterinary Sciences, University of Bologna, Ozzano dell’Emilia, Italy; 3 Unequi Ltd., West Bridgford, United Kingdom; 4 Equine Studies Group, Waltham Petcare Science Institute, Mars Horsecare, Leicestershire, United Kingdom

**Keywords:** horse, welfare, group housing, individual housing, feeding system

## Abstract

**Introduction:**

This study evaluated a novel Automatic Feeding Device (AFD) for hay provision in group-housed and individually housed horses.

**Methods:**

In Phase 1 (P1), behavioral responses to ground hay feeding (Control-Hay) and to hay delivery through the AFD were assessed in three group-housed mares under two conditions: AFD continuously open (O-AFD) and AFD alternating between open and closed periods (OC-AFD). In Phase 2 (P2), four individually housed horses were used to evaluate the behavioral effects of three AFD opening-closing schedules: long opening (LO-OP), medium opening (ME-OP), and short opening (SH-OP). Behavioral data were collected by continuous video recording. Data were analyzed using linear mixed-effects models, with treatment as fixed effect and horse as random effect (experimental unit). Principal component analysis was used to explore variability in agonistic behaviors among group-housed horses.

**Results and discussion:**

In P1, individual differences in foraging activity and agonistic behaviors were observed. Exploratory behaviors increased significantly when the AFD alternated between open and closed periods compared with when it remained open (3.93 ± 3.36 vs. 1.72 ± 3.86 min/h; *p* = 0.010). Compared with Control-Hay, which resulted in no residual hay, AFD delivery increased residual hay, averaging 3.98 ± 0.77 kg in O-AFD and 4.88 ± 1.00 kg in OC-AFD (*p* < 0.001). Social hierarchy strongly influenced access to the AFD in group-housed horses. In P2, foraging from the AFD declined progressively from LO-OP and ME-OP to SH-OP (46.91 ± 0.81, 42.80 ± 1.65, and 38.84 ± 0.89 min/h, respectively; *p* < 0.001). Stress-related behaviors directed toward the AFD were most frequent during SH-OP: pawing occurred up to seven to eight times more often than in LO-OP and ME-OP, while rubbing and teeth or tongue use were approximately two to three times higher. Residual hay increased progressively as access duration decreased (*p* < 0.001), accompanied by a slight reduction in hay intake rate. Behavioral responses to the AFD were influenced by social dynamics in group-housed horses and feeder access schedule in individually-housed horses. The medium opening interval (40 min open, 20 min closed) appeared to provide the most appropriate balance between regulating hay intake, maintaining feeding behavior and limiting stress-related behaviors.

## 1 Introduction

The domestic horse retains a feeding pattern similar to that of its feral counterpart, characterized in particular during daylight hours by prolonged bouts of slow forage intake alternated with short bouts of no intake in-between (trickle feeding). Under natural conditions, foraging can occupy up to 60–70% of daylight hours and 40–50% of the night, with non-feeding periods rarely exceeding more than 3 h in domesticated or semi-feral horses (1, 2). Stabling, especially associated with meal feeding and/or restricted forage provision often restricts the opportunity for many horses to express this natural feeding behavior (3). However, ad-libitum forage provision may not be optimal due to weight maintenance issues and therefore forage restriction is often required for health reasons (4, 5). Preserved forage is also commonly provided as loose hay on the ground (6), and it has been reported (7) that horses tend to consume loose hay more rapidly when they receive a limited number of rationed meals per day. Such practices often result in prolonged fasting periods between meals. Prolonged fasting periods adversely affect gastrointestinal health, predisposing horses to acidosis and gastric ulceration (8) and they can also impair mental wellbeing by promoting the development of stereotypic behaviors (9). In recent years, two main strategies have been developed to prolong hay intake and reduce fasting intervals: slowing down intake through the use of various slow feeders, and portioning forage into smaller, more frequent meals through the use of time-controlled hay racks that open and close at set intervals. Particularly, time-controlled hay racks are intended to allow more frequent feeding without increasing either the amount of hay ingested or the caretaker’s workload (10). In addition, automatic feeding systems may help reduce hay wastage compared to ground feeding (4) or free-choice forage provision (11) – an aspect of growing importance for both economic sustainability and stable hygiene.

For horses housed in groups, automatic systems have been developed to provide forage by granting access to the hay ration through individual transponder identification, such as pedometers or collar chips (12, 13). For horses housed individually, small automated feeders for complementary feed provision have been designed, mainly with the aim of reducing stereotypic behaviors such as cribbing (14) and health disorders such as gastric ulceration (15). However, the development of time-controlled systems for forage distribution in stalls seems more limited, likely due to spatial constraints, and no evidence-based recommendations currently exist regarding optimal opening and closing schedules for these devices. This highlights the need for further research to provide practical guidance for optimizing automated hay provision while supporting equine welfare.

The aim of the present study, therefore, was to evaluate the behavioral responses of group-housed (Phase 1) and individually housed horses (Phase 2) to a novel automatic feeding device (AFD) for hay provision. Specifically, the study investigated the influence of social dynamics on AFD use in group-housed horses, and the effects of different AFD opening and closing schedules on exploratory, feeding and stress-related behaviors in individually housed horses.

## 2 Materials and methods

### 2.1 Ethics statement

The study was approved by the Ethics Committee of the Department of Veterinary Sciences at the University of Turin (Protocol No. 0000391; 26/02/2024). All horses participating in the study remained in their usual home environment. Their care, housing, and daily management were the responsibility of their regular owner and grooms. Full consent from the owner was obtained prior to conducting the study.

### 2.2 Study design

The study consisted of two phases: the first phase (P1) used three group housed horses, followed by the second phase (P2) conducted on four individually housed horses. During each phase, horses’ intake and behavior was first assessed during their normal feeding routine (hay on ground) and then they were slowly introduced to the automatic feeding device (AFD) for 3 days (adaptation).

After the adaptation period, various opening and closing periods were introduced and evaluations were repeated 3 times for each AFD treatment schedule (P1: Open versus Open-Closed Schedule; P2: 4 × 4 Latin Square Design with open only to short to medium to long Open-Closed Schedules).

#### 2.2.1 The automatic feeding device

The automatic feeding device (AFD) used in the present study was developed based on a feeder previously employed by the research group in an earlier study (16). The AFD had the following dimensions of external dimensions (160 × 110 × 100 cm) and internal dimensions of 106 × 86 × 60 cm, corresponding to an internal volume of approximately 0.55 m^3^ and a hay-holding capacity of about 16–20 kg. The feeder was a mobile unit mounted on four wheels and equipped with a control unit for automated programming. All edges of the device were covered with a rubber sheath to ensure the horse’s safety. The system operated by connecting to a power source and featured a sliding-door mechanism located at the upper part of the device, which controlled the horse’s access to the hay.

The automatic operation was programmable, allowing the AFD to remain open or closed for adjustable intervals ranging from 1 s to 99 min. Each time the door opened or closed, a soft audible signal was emitted to indicate activation, though it did not create any issues to the animals beyond a slight concern during the initial acclimation (this feature could be muted). It was also possible to set the operating period within a 24-h cycle. Outside the selected time window, the feeder remained closed. The AFD was designed to allow simultaneous access from three sides and could therefore accommodate up to three horses at the same time.

#### 2.2.2 Animals

All the horses had been routinely monitored by their attending veterinarian and were reported to be in good overall health. Prior to the start of the trial, the horses underwent a dental check during which no dental issues were found and the horses received a dental score (17) of 0.5 ± 0.5 (mean ± SD).

##### 2.2.2.1 Phase 1: group housed horses

Phase 1 utilized three adult female horses, identified as Blue, White, and Green, aged 22 ± 5.57 years (mean ± SD), with a mean ±SD Body Condition Score (BCS) of 5.5 ± 1.5 on a 1–9 scale (18), and a mean ±SD body weight (BW) of 540 ± 188 kg. The horses had lived together for the past 6 years in a dry-lot paddock with access to a 6 m × 8 m shelter with rice-husk bedding. They were routinely fed long-stem first-cut hay, provided on the ground in three separate corners of the shelter. Hay was distributed in four daily meals (7 a.m., 12 p.m., 5 p.m., 9 p.m.), providing a minimum daily hay availability equivalent to 1.5% of BW on a dry matter (DM) basis (19).

The AFD was placed inside the 6 m × 8 m shelter and horses could access the hay AFD from 3 sides (Figure 1).

**Figure 1:**
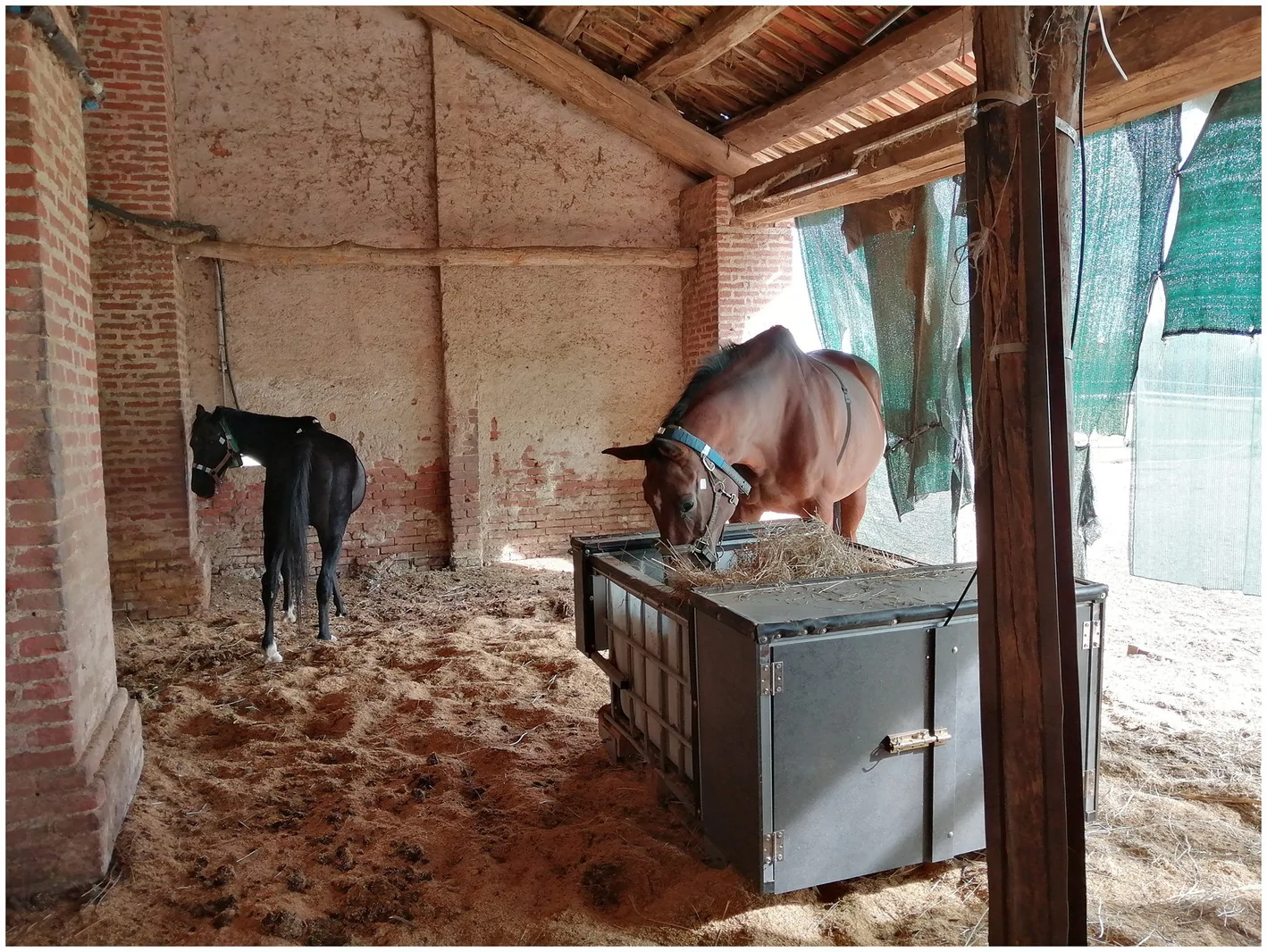
The automatic feeding device (AFD) used to feed the group housed horses.

##### 2.2.2.2 Phase 2: individual housed horses

Phase 2 involved a different cohort of four adult horses, two mares and two geldings, aged 20.3 ± 1.3 years, with mean ± SD BCS of 6.5 ± 1 on a 1–9 scale (18), and a mean ±SD BW of 500 ± 88.4 kg. The horses were housed in individual boxes adjacent to each other, allowing visual contact. Each horse had daily access to individual dry lot paddocks for approximately 4 h per day. The horses were fed, according to the management routine of the equestrian center, a long stem meadow hay loose on the ground as three meals per day at the following times: 7 a.m., 12 p.m., and 6 p.m., ensuring a minimum daily hay availability equivalent to 1.5% of BW, calculated on a DM basis (19). In addition, during the morning meal they were fed 1 kg of complementary feed (labeled to contain crude protein 13%, ether extract 6%, crude fiber 9%, ash 8.50%; as fed).

During the feeding trial carried out on the single housed horses, the AFD was placed in front of the horse’s stall entrance. The stall door was kept open, and a stall guard separated the horse from the device. The horse could access the feeder only from its longitudinal side, which was equipped with an additional wooden protection panel (Figure 2).

**Figure 2:**
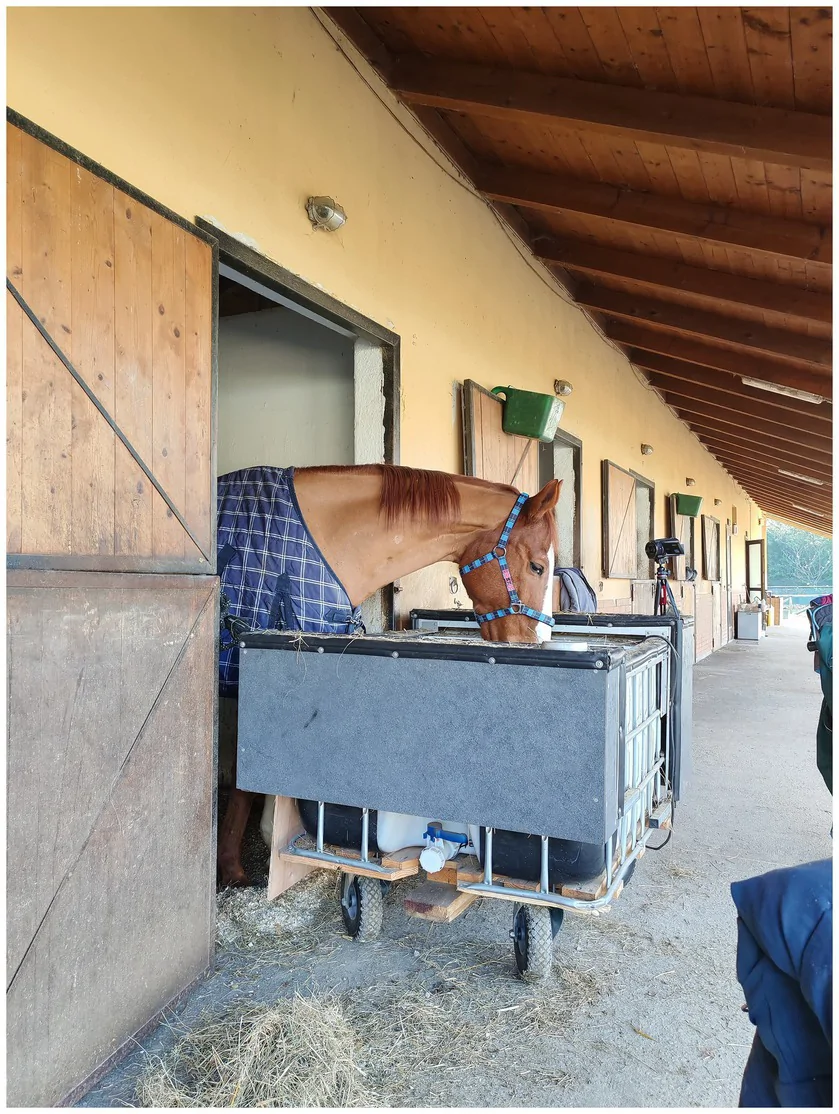
The automatic feeding device (AFD) used to feed the individually housed horse.

### 2.3 Feeding schedule

All horses were adapted to be fed from the novel automatic feeding device (AFD) during a 3-day adaptation period. The adaptation period consisted of incremental exposure to the AFD starting from exposure to the empty AFD on Day 1, followed by access to the AFD filled with hay but kept permanently open on Day 2 and Day 3. The adaptation concluded with the activation of the mechanism for 10 min at the end of Day 3, performing three open-close cycles (each consisting of a 2-min opening phase and a 1-min closed phase) to adapt the horses to the noise and movement of the AFD.

#### 2.3.1 Phase 1: group housed horses

After the adaptation period, the group-housed horses were exposed to three experimental feeding conditions: Ground-feeding condition (Control-Hay), AFD open condition (O-AFD), and AFD open/close condition (OC-AFD). Each feeding treatment was evaluated for two consecutive days, with one experimental feeding section conducted per day, between 08:30 and 12:30, for an average duration of 3 ± 1 h. A single AFD was available to the group.

(1) Ground-feeding condition (Control-Hay): a total of 10 kg long-stem hay was provided to the group of three horses on the ground, distributed in three separate piles inside the shelter, with individual quantities adjusted proportionally to body weight as per their usual routine. The AFD was placed inside the shelter, open but without hay, positioned along one of the shorter sides.
(2) AFD open (O-AFD) condition: a total of 10 kg of hay was placed inside the AFD, which was placed in the center of the shelter. The automatic mechanism was not activated, and the device remained open throughout the entire feeding session.
(3) AFD open/close (OC-AFD) condition: a total of 10 kg of hay was placed inside the AFD, which was in the center of the shelter. The automatic opening/closing function was activated, with cycles of 30 min open followed by 45 min closed. This sequence was repeated for three full cycles, for a total session duration of 3 h and 45 min.

In both AFD conditions (2 and 3), the 10 kg of hay provided within the AFD represented the sole source of forage available during the feeding trial; no additional hay was provided. Outside of these experimental hours, the horses were managed and fed by their owners according to their usual routine (see section 2.2.2.1).

#### 2.3.2 Phase 2: individual housed horses

After the horses were adapted to the AFD, a Latin Square Design 4 × 4 was used, in order for each horse to be fed the same hay by each of the four feeding methods:

(1) 4 kg of hay on the ground (Control-Hay).
(2) 4 kg of hay inside the AFD with time-set of 60 min open and 15 min closed, repeated for 2 full cycles in a total trial time of 150 min (long opening period LO-OP), resulting in a total hay availability of 120 min (Figure 3).
(3) 4 kg of hay inside the AFD with time-set of 40 min open and 20 min closed, repeated for 2 full cycles, followed by a final 30 min opening period in a total trial time of 150 min, resulting in a total hay availability of 110 min (medium opening period ME-OP) (Figure 3).
(4) 4 kg of hay inside the AFD with time-set of 25 min open and 15 min closed, repeated for 4 full cycles, ending with a final 5 min closure in a total trial time of 150 min, resulting in a total hay availability of 100 min (short opening period SH-OP) (Figure 3).

**Figure 3:**
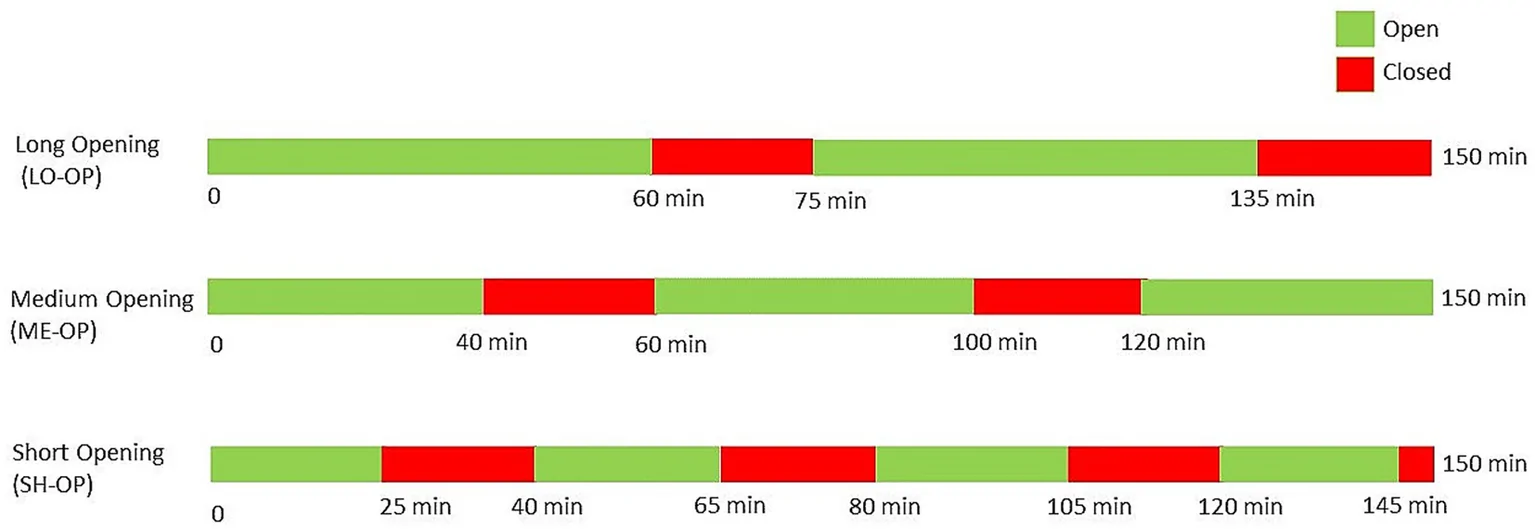
Timeline of the AFD feeding protocols during the 150-min trial. Green and red bars represent hay access (Open) and restriction (Closed) periods, respectively. Long opening period LO-OP: 60 min open and 15 min closed; medium opening period ME-OP: 40 min open and 20 min closed; short opening period SH-OP 25 25 min open and 15 min closed.

The AFD was used only during the experimental 150-min feeding session. Outside the observation period, horses were managed and fed according to the routine practices of the equestrian center (as described in section 2.2.2.2).

The hay provided during the entire study was the same type routinely fed to the horse: a long-stem, first-cut meadow hay. A single batch of this hay was used throughout the study.

At the end of each observation period, all leftover hay, including hay remaining in the feeder and hay dropped onto the ground, was collected and weighed to determine residual hay. In the AFD treatments, residual hay included both hay remaining inside the AFD and hay dropped onto the ground, whereas in the Control-Hay treatment it included all uneaten hay remaining in the feeding area. Hay intake was calculated as the difference between the amount of hay offered and the residual hay recovered.

### 2.4 Video recording and analysis

For group-housed horses, two Sony HDR-CX240 video cameras with 1080p HD resolution were used. One camera was positioned overhead and the other laterally, without interfering with the animals’ daily activities. Both cameras were mounted on tripods and placed to provide the most complete view possible of the horses and the feeding device. The cameras recorded the entire shelter area where the animals were housed for approximately 3 ± 1 h per day.

For individually housed horses, each horse was video-recorded during the morning and the midday meals with a 2D camera (Sony HDR-CX240 cameras with 1080p HD resolution). The camera was placed on a tripod outside the box at the same distance of 0.9 m for each horse, positioned to record the animal in profile for the entire duration of the trial. Each recording lasted 150 min, corresponding to the duration of the feeding session. At the beginning and end of each recording, the start and end times were displayed on camera to ensure completeness and accuracy of timing.

The videos were analyzed using a continuous sampling method with BORIS, an open-source software for event logging and behavioral video coding [Behavioral Observational Research Interactive Software, version v.8.20, University of Turin, Italy (20)], by the same trained operator experienced in identifying equine behaviors. Behaviors were categorized as described in Table 1.

**Table 1:** Description of the behaviors analyzed.

Behavioral category	Behavioral label	Definition	Measure type	Applicability	Illustration
Exploring behaviors	Approaching the AFD	Approach the AFD (>2 m)	Time-based behaviors, minutes/h	Group-housed horses	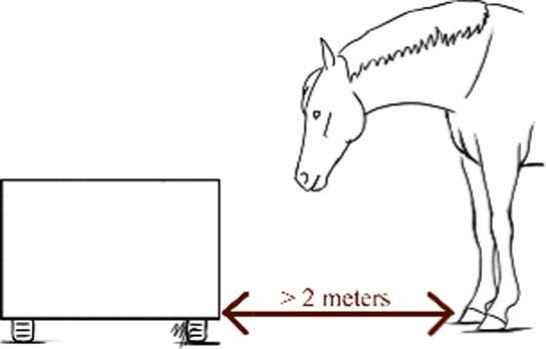
	Exploring the AFD	Exploring the AFD (<2 m), nose below the belly line, ears and eyes directed toward the object	Time-based behaviors, minutes/h	Group-housed horses Individual housed horses	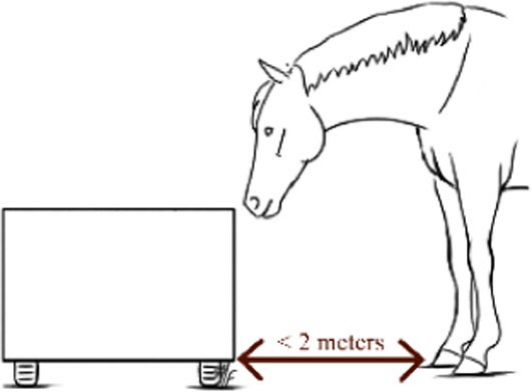
	Sniffing the AFD content	The horse sniff the AFD content	Count-based behaviors, counts/h	Group-housed horses Individual housed horses	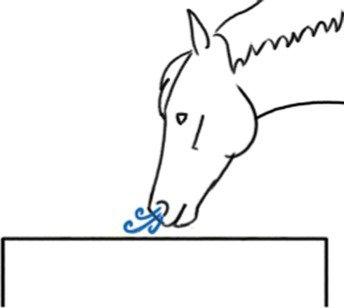
Feeding behaviors	Foraging from AFD	Horse bites and chews forage from the AFD	Time-based behaviors, minutes/h	Group-housed horses Individual housed horses	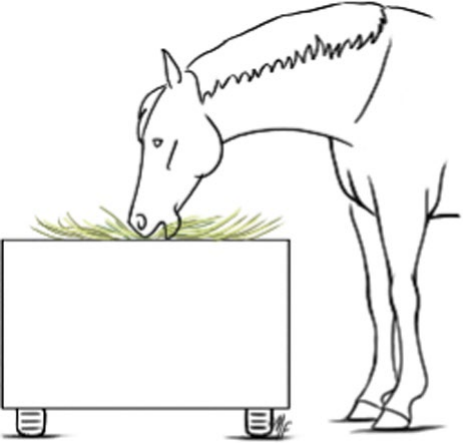
	Foraging on the ground	Horse bites and chews forage on the ground	Time-based behaviors, minutes/h	Group-housed horses Individual housed horses	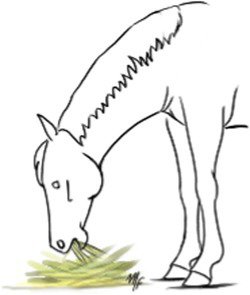
Stress-related behaviors toward the AFD	Fear reaction	Head raised, tense posture, and focused attention toward the feeding device	Count-based behaviors, counts/h	Group-housed horses Individual housed horses	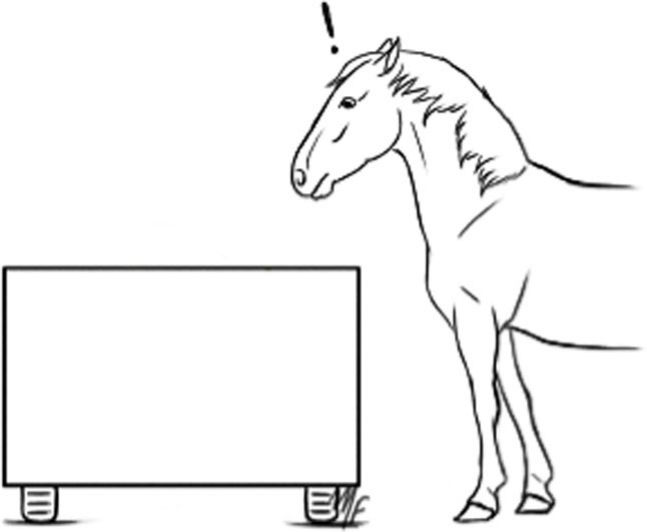
	Rubbing the head on AFD	The horse rubs its head or muzzle against the feeding device.	Time-based behaviors, minutes/h	Group-housed horses Individual housed horses	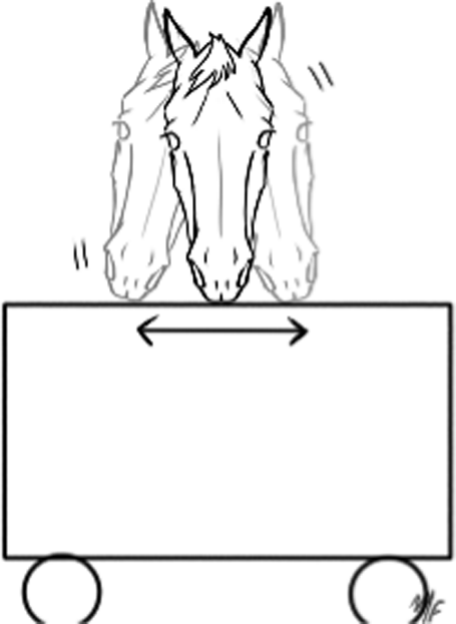
	Pawing	The horse paws the ground or strikes the feeding device with a foreleg.	Count-based behaviors, counts/h	Group-housed horses Individual housed horses	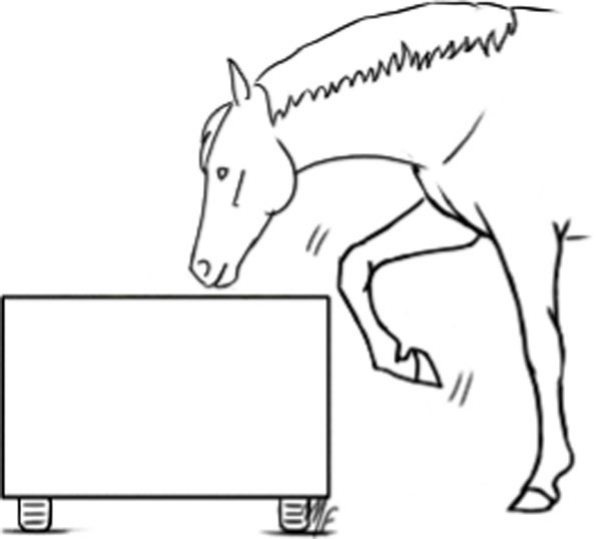
	Using the teeth on AFD	The horse uses its teeth to bite or grasp the feeding device.	Count-based behaviors, counts/h	Individual housed horses	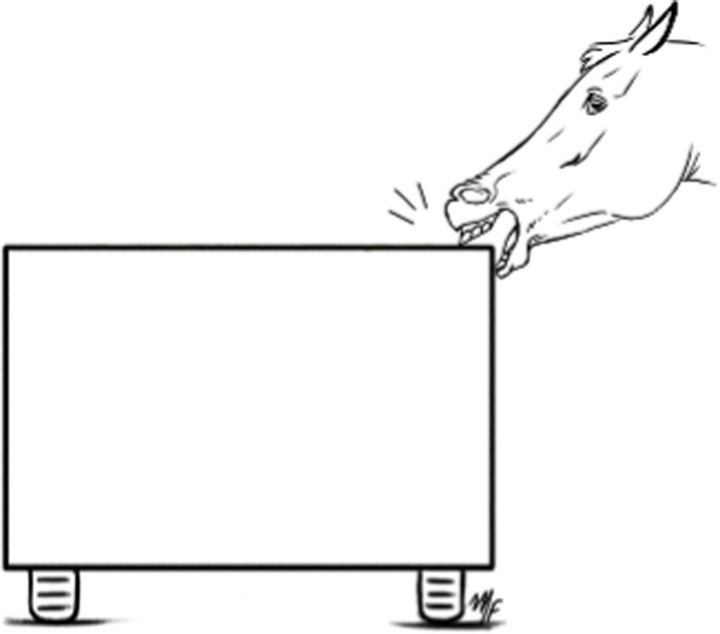
	Using the tongue on AFD	The horse licks the surface of the feeding device with its tongue.	Time-based behaviors, minutes/h	Individual housed horses	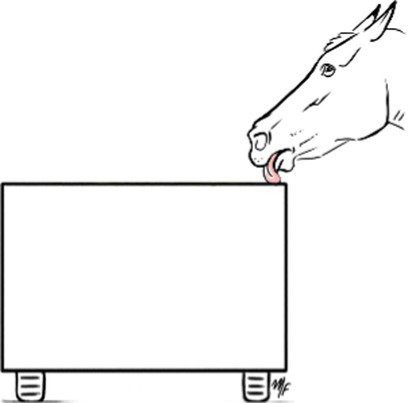
Social behaviors	Displacement given	Interaction directed toward another individual, with the head outstretched and ears flat back against the head resulting in recipient raising head, or taking a step away in any direction	Count-based behaviors, counts/h	Group-housed horses	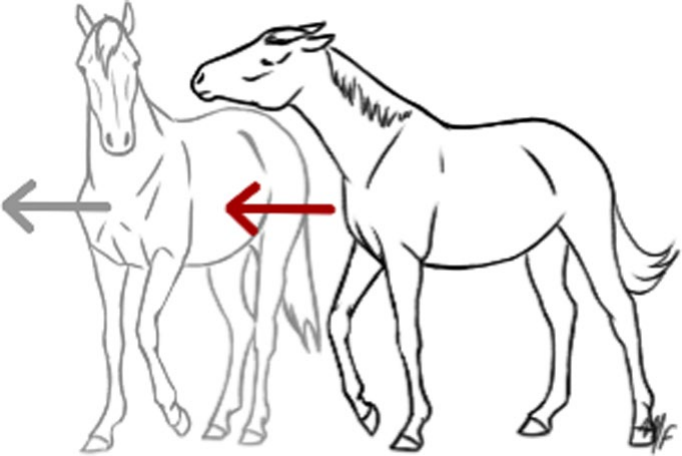
	Displacement received	Interaction received from another individual defined as above, causing recipient to raise head, move sideways the body or just the neck or take a step away	Count-based behaviors, counts/h	Group-housed horses	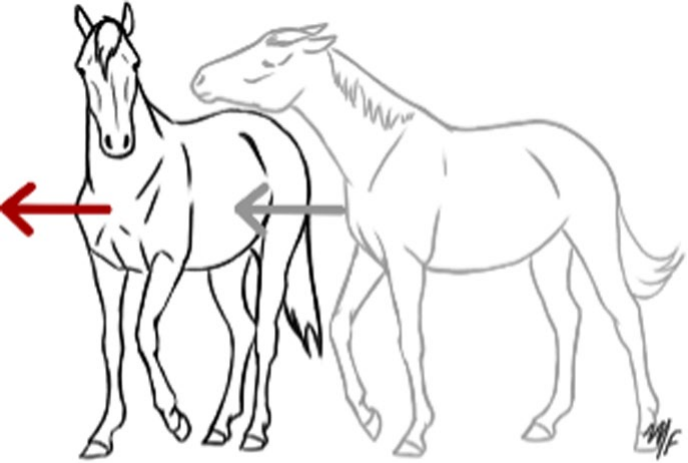
	Bite another horse	The horse quickly opens and closes its mouth and its teeth touch or not the body of a conspecific, aiming to bite him. The ears are turned backwards	Count-based behaviors, counts/h	Group-housed horses	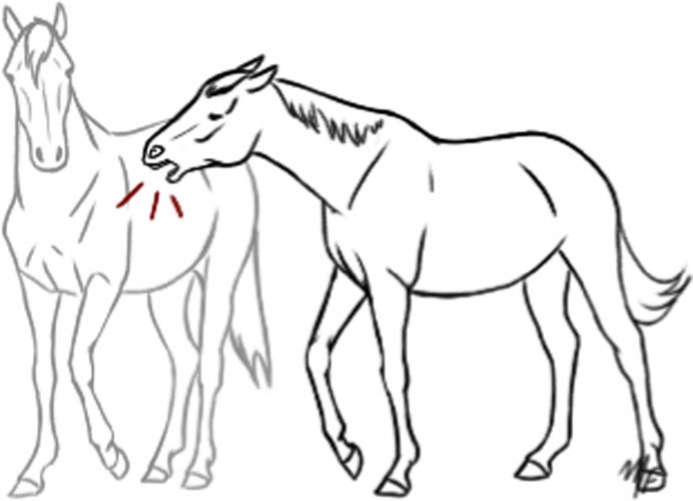
	Kicking another horse	The horse lifts one or both hind limbs off the ground and quickly stretches it/them toward a conspecific, aiming to hit him	Count-based behaviors, counts/h	Group-housed horses	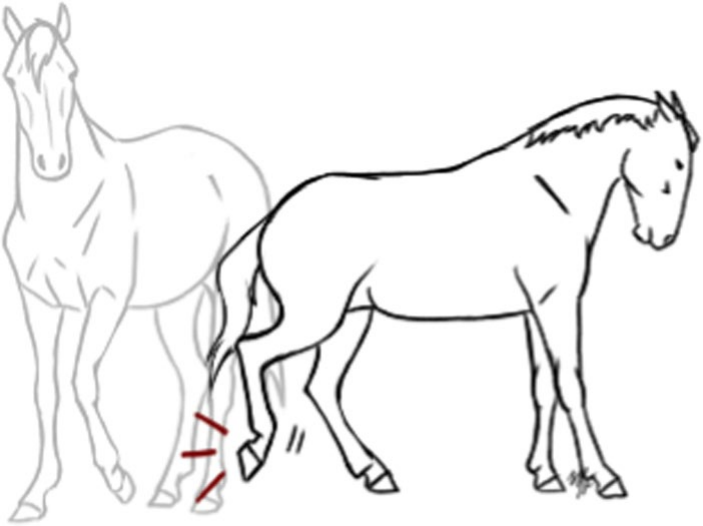
	Calling at conspecifics	The horse makes vocalization to call conspecifics	Count-based behaviors, counts/h	Group-housed horses Individual housed horses	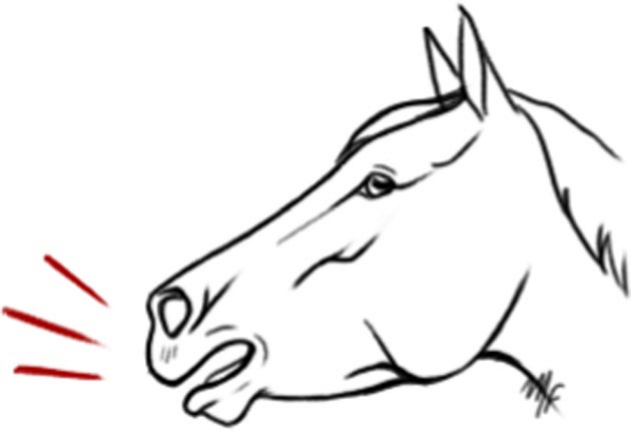

### 2.5 Statistical analysis

Statistical analyses were performed using a freeware statistical software package (JMP Pro, Version 19; SAS Institute Inc., Cary, NC, USA). Normality of each variable was assessed with the Shapiro–Wilk test, and non-normal variables were normalized when necessary, using a Box–Cox transformation. A linear mixed-effects model was constructed, with AFD group as the fixed effect and horse as the random effect (experimental unit). Each measurement was treated as a repeated observation. When the model showed significant effects, Tukey’s post-hoc test was used to differentiate means. Considering group housed horses, to further investigate individual differences in AFD use, a second linear mixed-effects model was constructed for total time spent foraging, including Horse (Blue, White and Green), Feeding condition, and their interaction (Horse × Feeding condition) as fixed effects. In addition, a principal component analysis (PCA; correlation matrix) was used to investigate variability in agonistic behaviors of group-housed horses in relation to individual horses (Blue, Green and White) and AFD feeding schedules (AFD open and AFD open/close). Considering individual housed horses, a linear regression analysis and Pearson’s correlation coefficient were performed to evaluate the relationship between time spent foraging when the AFD was open (LO-OP 120 min, ME-OP 110 min, SH-OP 100 min) and hay intake (kg). The significance level was set at *p* < 0.05.

## 3 Results

### 3.1 Phase 1: group housed horses

The use of the Automatic Feeding Device (AFD) in group-housed horses showed no significant differences between AFD open and AFD open/close in the time spent approaching the device, nor in the frequency of sniffing its contents. Fear-related responses and social behaviors, including displacements given or received, biting, and kicking, did not vary across Control-Hay and AFD conditions (AFD open and AFD open/close).

Significant effects between AFD open and AFD open/close were observed for exploratory interactions with the device (Table 2): horses engaged in more exploration when the AFD was in open/close condition compared to when it was continuously open (3.93 ± 3.36 min/h vs. 1.72 ± 3.86 min/h; *p* = 0.010). Rubbing the head on the device occurred almost exclusively in the open/close condition and was significantly higher than in the open condition (0.49 ± 0.56 min/h vs. 0.03 ± 0.05 min/h; *p* < 0.001).

**Table 2:** Mean (± standard deviation) behaviors for feeding from the ground (Control-Hay) or from the automatic feeding device (AFD) according to the different feeding methods – Open AFD (O-AFD), open/close AFD (OC-AFD).

Behavior	Control-Hay	O-AFD	OC-AFD	*p*-value
Approaching the AFD, min/h	NA	0.32 ± 0.25	0.23 ± 0.17	0.398
Exploring the AFD, min/h	NA	1.72 ± 3.86	3.93 ± 3.36	0.010
Sniffing the AFD content, count/h	NA	1.75 ± 1.08	1.43 ± 1.84	0.374
Foraging from AFD, min/h	NA	30.84 ± 20.34	14.06 ± 10.96	0.100
Foraging on the ground, min/h	36.71 ± 8.96^A^	13.27 ± 9.65^B^	16.64 ± 6.55^B^	<0.001
Total time spent foraging, min/h	36.71 ± 8.96^B^	44.11 ± 14.25^A^	30.70 ± 10.08^B^	0.003
Fear reaction, count/h	NA	0.25 ± 0.28	0.08 ± 0.19	0.131
Rubbing the head on AFD, min/h	NA	0.03 ± 0.05	0.49 ± 0.56	<0.001
Pawing, count/h	0.00 ± 0.00	0.05 ± 0.11	0.03 ± 0.08	0.581
Displacement given, count/h	2.83 ± 2.45	6.05 ± 8.40	4.49 ± 5.86	0.717
Displacement received, count/h	3.04 ± 2.77	5.47 ± 4.19	4.56 ± 3.43	0.093
Bite another horse, count/h	0.08 ± 0.19	0.85 ± 1.28	0.50 ± 0.67	0.142
Kicking another horse, count/h	0.25 ± 0.56	0.27 ± 0.47	0.00 (0.00)	0.161
Calling at conspecific, count/h	0.00 ± 0.00	0.00 ± 0.00	0.00 ± 0.00	1.000

Data are expressed as mean ± standard deviation (SD). Different superscript letters within a row indicate significant differences (*p* < 0.05).

NA, not applicable.

The time spent foraging from the ground during the AFD trials did not differ between conditions (O-AFD: 13.27 ± 9.65 min/h and OC-AFD: 16.64 ± 6.55 min/h). However, the total time spent foraging was higher in the O-AFD condition (44.11 ± 14.25 min/h) compared to Control-Hay (36.71 ± 8.96 min/h) and OC-AFD (30.70 ± 10.08 min/h), whereas no difference was observed between Control-Hay and OC-AFD. The amount of residual hay differed among ground versus AFD treatments (*p* < 0.001): no leftover hay was observed in the Control-Hay condition, whereas horses left (mean ± SD) 3.98 ± 0.77 kg of hay when fed from the AFD left open and 4.88 ± 1 kg in the open/close condition.

The PCA biplot (Figure 4) showed a clear separation among group-housed horses. The Blue horse was associated with higher values of total foraging time and with a greater frequency of social behaviors directed toward conspecifics, including displacement given, biting, and kicking. In contrast, the White and Green horses were associated with lower foraging activity and higher frequencies of displacement received. Table 3 shows that the foraging time differed significantly among horses (*p* < 0.001), with the Blue and White horses spending more time foraging than the Green horse (44.18 ± 7.51, 38.31 ± 12.03 and 24.18 ± 4.87 min/h, respectively). Foraging time also differed between feeding conditions (*p* = 0.002), whereas the Horse × Feeding condition interaction was not significant (*p* = 0.153), suggesting that horses differed in their foraging behavior regardless of the feeding treatment applied. Moreover, aggressive and dominance-related behaviors clustered primarily according to individual horses rather than by feeding conditions (feeding on the ground, AFD open and AFD open/close), indicating an individual (horse) effect than an AFD treatment effect.

**Figure 4:**
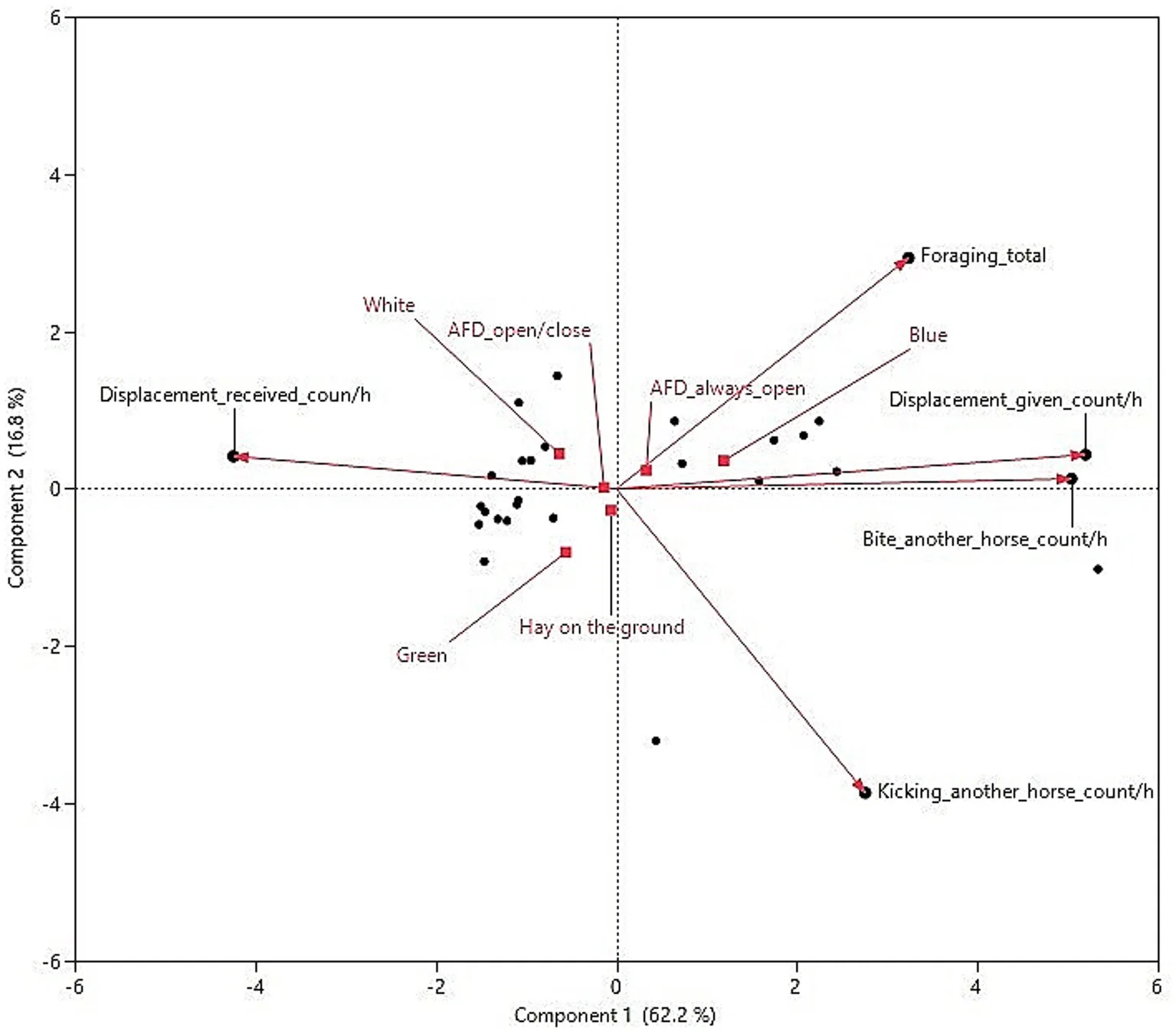
Principal component analysis biplot (PC1 and PC2) performed on foraging behavior and social behaviors (displacement given, displacement received, bite another horse, kicking another horse) in group-housed horses (White, Green and Blue).

**Table 3:** Foraging time (min/h) under the three feeding conditions (Control-Hay, O-AFD and OC-AFD) and total time spent foraging (min/h) of each group-housed horse (Blue, White and Green).

Feeding condition	Horses			*p*-value		
	Blue	White	Green	Horse	Feeding condition	Horse × Feeding condition
Foraging from the ground (Control-Hay), min/h	43.85 ± 7.99	39.69 ± 3.26	26.60 ± 1.56	<0.001	0.002	0.153
Foraging from open AFD (O-AFD), min/h	51.30 ± 7.78	54.37 ± 5.14	26.66 ± 1.56			
Foraging from open/close AFD (OC-AFD), min/h	40.78 ± 6.40	29.58 ± 7.11	21.74 ± 6.04			
Total time spent foraging (min/h)*	44.18 ± 7.51^A^	38.31 ± 12.03^A^	24.18±4.87^B^			

Data are expressed as mean ± standard deviation (SD).

*The overall mean total time spent foraging (min/h) differs from the means of the individual feeding conditions (Control-Hay, O-AFD and OC-AFD) due to the different numbers of observations across feeding conditions.

Significant differences (*p* < 0.05).

### 3.2 Phase 2: individual housed horses

Feeding behavior was influenced by the programmed opening time. Exploratory, feeding, and stress-related behaviors toward the AFD varied according to the feeding method (Table 4). In particular, the duration of foraging from the AFD decreased progressively from LO-OP (46.91 ± 0.81 min/h) to ME-OP (42.80 ± 1.65 min/h) to SH-OP (38.84 ± 0.89 min/h) (*p* < 0.001), while the time spent foraging on the ground showed the opposite trend, being highest in the Control-Hay condition and lowest in LO-OP. This shift in feeding patterns was also reflected in the amount of residual hay, which increased as the opening period shortened (*p* < 0.001). Specifically, horses left (mean ± SD) 0.18 ± 0.08 kg/150 min in Control-Hay, 0.82 ± 0.55 kg/150 min in LO-OP, 1.14 ± 0.61 kg/150 min in ME-OP, and 1.49 ± 0.68 kg/150 min in SH-OP (*p* < 0.001). This pattern is consistent with the direct measurements of hay intake (kg/h), which progressively decreased from Control-Hay (1.53 ± 0.03) to LO-OP (1.27 ± 0.22), ME-OP (1.14 ± 0.24), and SH-OP (1.00 ± 0.27) (*p* < 0.001). Moreover, Table 5 shows the mean foraging behavior and hay intake according to the different methods in relation to the time when the AFD was open (LO-OP 120 min, ME-OP 110 min, SH-OP 100 min). Figure 5 represents graphically the linear regression between foraging when the AFD was open and hay intake (kg). Linear regression analysis and Pearson’s correlation coefficient reveal a significant positive relationship between AFD-open foraging time and hay intake (*r* = 0.67, *R*^2^ = 0.44, *p* < 0.001), indicating that hay intake increased with increasing AFD-open foraging time. The model explained 44% of the variability in hay intake, while the remaining variation was likely attributable to inter-individual differences in feeding behavior. Horses spent a similar amount of time exploring the feeding device across the automated feeding treatments. Stress-related behaviors were generally more frequent when the AFD operated with shorter opening intervals. Horses exhibited significantly more head-rubbing, pawing, and use of the teeth and the tongue on the AFD in the SH-OP condition compared to LO-OP and ME-OP.

**Table 4:** Mean behaviors and feed intake (± standard deviation) according to different feeding methods (AFD, Automatic feeding device, Control-Hay: Hay on ground, AFD: LO-OP long opening, ME-OP medium opening, SH-OP short opening).

Behavior	Control-Hay	LO-OP	ME-OP	SH-OP	*p*-value
Exploring the AFD, min/h	NA	0.44 ± 0.50	0.38 ± 0.34	0.36 ± 0.26	0.928
Sniffing AFD content, count/h	NA	0.85 ± 1.00	0.43 ± 0.66	0.6 ± 0.67	0.199
Foraging from AFD, 150 min*	NA	117.27 ± 1.97^A^	107.01 ± 4.12^B^	97.10 ± 2.22^C^	<0.001
Foraging from AFD, min/h*	NA	46.91 ± 0.81^A^	42.80 ± 1.65^B^	38.84 ± 0.89^C^	<0.001
Foraging on the ground, min/h	43.57 ± 2.76^A^	0.43 ± 0.48^C^	1.44 ± 1.33^B^	2.20 ± 1.56^B^	<0.001
Hay intake, kg/h*	1.53 ± 0.03^A^	1.27 ± 0.22^B^	1.14 ± 0.24^C^	1.00 ± 0.27^D^	<0.001
Fear reaction, count/h	0.00 ± 0.00^B^	1.30 ± 0.36^A^	1.28 ± 0.57^A^	2.23 ± 1.46^A^	<0.001
Rubbing head on AFD, min/h	NA	0.06 ± 0.08^B^	0.05 ± 0.06^B^	0.21 ± 0.23^A^	<0.001
Pawing, count/h	0.00 (0.00)^B^	0.38 ± 0.93^B^	0.43 ± 0.89^B^	3.00 ± 4.94^A^	<0.001
Using the teeth on AFD, count/h	NA	2.73 ± 5.44^B^	3.95 ± 6.92^B^	6.25 ± 9.63^A^	<0.001
Using the tongue on AFD, min/h	NA	0.10 ± 0.22^AB^	0.03 ± 0.05^B^	0.29 ± 0.56^A^	<0.001
Calling at conspecific, count/h	0.00 ± 0.00	0.00 ± 0.00	0.00 ± 0.00	0.05 ± 0.14	0.133

Different superscript letters within a row indicate significant differences (*p* < 0.05).

NA, not applicable.

* Foraging from the AFD and hay intake are expressed as minutes per hour over the total observation period (150 min), including both when the AFD was open and when it was closed.

**Table 5:** Mean (±SD) foraging behavior (min) and hay intake (kg) according to the different methods in relation to the time during which the AFD was open (LO-OP 120 min, ME-OP 110 min, SH-OP 100 min).

Behavior	Foraging from AFD open (min)	Hay intake (kg)
LO-OP, 120 min open	93.81 ± 1.57	2.55 ± 0.43
ME-OP, 110 min open	78.47 ± 3.02	2.09 ± 0.45
SH-OP, 100 min open	64.73 ± 1.48	1.67 ± 0.45

**Figure 5:**
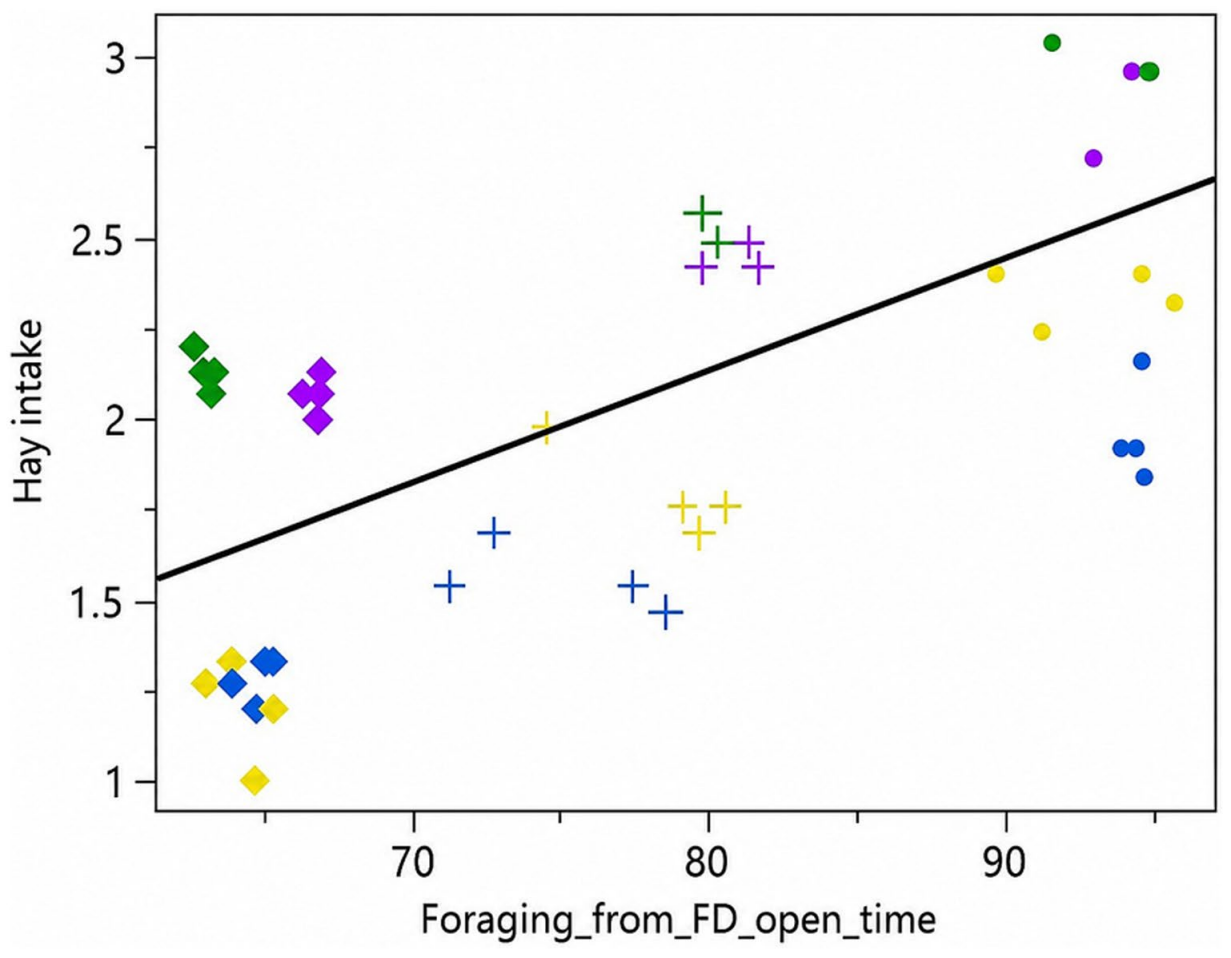
Linear regression analysis of the relationship between foraging when the AFD was open (LO-OP 120 min, ME-OP 110 min, SH-OP 100 min) and hay intake (kg). Diamonds (♦) represent the SH-OP feeding method, crosses (+) represent the ME-OP feeding method, and dots (•) represent the LO-OP feeding method. Different colors indicate the different horses.

## 4 Discussion

The present study investigated the behavioral responses of both group-housed and individually housed horses to a newly developed Automatic Feeding Device (AFD) designed to provide long-stem hay in a way to optimize both health and welfare.

In group-housed horses, the PCA biplot (Figure 4) highlighted individual differences in social interactions and foraging activity. In particular, the Blue horse aligned with higher total foraging time and higher frequencies of displacements given, biting and kicking, whereas the White and Green horses aligned with higher displacements received and lower foraging activity. Importantly, agonistic behaviors clustered primarily by individual horse rather than by feeding condition (AFD open vs. AFD open/close), suggesting that interactions around the feeder were more strongly driven by horse-dependent (likely dominance-related) differences than by the device operating mode. These findings are consistent with those reported by Baumgartner et al. (21), who showed that competition and stress at time-controlled hay racks in group-housed horses are strongly influenced by the spatial organization of the feeding system, particularly the animal-to-feeding-place ratio. In their study, aggressive behaviors such as biting and kicking were markedly reduced only when at least three feeding places per horse were provided. Similarly, Seabra et al. (11), reported higher frequencies of agonistic behaviors, including biting and kicking, in groups of five horses fed through an automatic box feeder compared with free-choice forage provision, suggesting that restricted access to forage may increase competition around the feeding resource. However, as the three horses in the present study had been living together for a long time (6 years) such a strong hierarchical impact was not anticipated. These findings underline that, when feeding horses in groups, hierarchy, social cohesion and the availability of sufficient space and escape routes are critical to minimize the risk of escalated competition at feeding sites (22). Some automatic forage feeders designed for open paddock (21) include solid barriers between access points to reduce competition. The current feeding device potentially could be used in a small group of horses without a major hierarchical discrepancy, but caution would be needed otherwise. In the present study, foraging time differed among group-housed horses, whereas the Horse × Feeding condition interaction was not significant, indicating that individual differences in foraging behavior were maintained across all feeding treatments (Table 3). This suggests that some horses consistently achieved greater access to forage than others regardless of feeder operating mode. Although long-term individual forage intake, body condition and health outcomes were not assessed in the present study, such persistent differences in feeder use could have important implications under practical management conditions. In particular, unequal access to forage may contribute to differences in nutrient intake, body condition and welfare among group-housed horses, especially where strong dominance relationships exist.

Exploratory behavior toward the device increased when the AFD operated with intermittent opening and closing cycles, accompanied by a rise in head-rubbing behavior. This pattern may reflect both curiosity and anticipatory behavior, a motivational state that arises when animals predict the delivery of a desired resource (23). Importantly, fear-related responses remained rare across all conditions, indicating that neither the movement nor the noise of the mechanism induced avoidance. Ground foraging, defined as hay extracted from the AFD, dropped, and subsequently consumed from the ground, was substantially reduced in both AFD conditions (open and open/close) compared to the control condition, as expected when hay is no longer supplied freely on the ground. Interestingly, this observation differs from the findings of Seabra et al. (11), who reported increased ground-oriented behaviors in horses managed with an automatic box feeder. However, the behaviors evaluated were not directly comparable. In the present study, ground foraging referred specifically to the consumption of hay that had been extracted from the feeder and subsequently eaten from the ground, whereas Seabra et al. (11) included behaviors such as ground sniffing and coprophagy, which are more indicative of food-searching activity. Furthermore, the feeding schedules differed substantially between studies, as their feeder provided six 60-min feeding opportunities distributed throughout the day (i.e., 60 min open and 180 min close), resulting in longer intervals without forage access between feeding opportunities. In contrast, the present study AFD operated with much shorter closure periods, which may have reduced food-searching behavior. The current study provided an initial assessment of frustration and intake behaviors in relation to timings but would need to be rolled out to long term observations to be fully comparable to Seabra et al. (11).

Finally, time spent foraging from the AFD did not differ between the open and open/close conditions, suggesting that horses compensated for closed intervals by increasing intake during open phases. This interpretation is supported by previous work demonstrating the ability of horses to adapt their feeding behavior when access to forage is temporarily restricted. Ince et al. (24), reported that ponies with ad libitum access to haylage but limited daily access to pasture progressively increased their intake during the available grazing period over several weeks, while maintaining a similar estimated total daily dry matter intake. Although the time scale and feeding conditions differed from those of the present study, these findings suggest that horses are capable of adjusting short-term intake patterns to compensate for periods of restricted forage access.

The results from individually housed horses provide further insight into how access duration affects feeding behavior. Over the entire observation period (150 min, including both AFD open and closed phases), longer open periods (LO-OP: 60 min open and 15 min close) were associated with a higher expression of foraging behavior from the AFD and a lower amount of leftover hay. The ME-OP condition (40 min open and 20 min close) showed an intermediate pattern, with slightly reduced foraging time and a modest increase in residual hay.

The difference in foraging behavior between the group-housed and individually housed horses likely reflects the different social and environmental contexts in which the AFD was used. Caution is required when considering the findings from the group-housed and individually housed trials together, as the two phases differed substantially in their environmental and management conditions. The group-housed trial was specifically designed to evaluate how horses interacted with a shared AFD under social conditions, whereas the individual-housing trial was designed to assess the behavioral effects of different opening and closing schedules when horses had unrestricted access to the feeder. Therefore, differences between phases therefore extend beyond housing type and include the number of horse-to-feeder ratio, feeder access opportunities, forage allocation, and feeding schedules. Consequently, the results should not be interpreted as a direct comparison between group and individual housing systems.

In the group-housed horses, access to the feeder was influenced by social dynamics, which likely affected individual opportunities to forage. In contrast, individually housed horses had unrestricted individual access to the AFD, allowing the effect of opening duration to emerge more clearly. Under these conditions, progressively shorter opening periods resulted in a proportional reduction in foraging time and hay intake. Indeed, shorter access cycles (SH-OP: 25 min open and 15 min close) elicited the highest levels of frustration-related behaviors—such as pawing, head rubbing, and the use of the teeth on the device—along with the lowest foraging duration and the greatest amount of residual hay. This behavioral pattern was further supported by quantitative measures of hay intake. Although all treatments provided the same total hay availability, intake rate (kg/h) decreased progressively with shorter access durations, from 1.53 ± 0.03 in Control-Hay to 1.27 ± 0.22 in LO-OP, 1.14 ± 0.24 in ME-OP, and 1.00 ± 0.27 in SH-OP (*p* < 0.001). When considering only the periods during which the AFD was open, this pattern was further supported by linear regression and Pearson’s correlation analysis conducted on AFD-open foraging time (see Table 5; Figure 4), confirming that, not perhaps surprisingly, increased access time was associated with higher hay consumption.

These findings align with literature reporting that decreasing foraging access progressively increases behavioral signs of feeding frustration (9). The opening and closing schedules evaluated in the present study were selected to provide short, medium and long forage access periods. In particular, the medium opening schedule (40 min open, 20 min closed) was based on previous observations showing that ponies fed forage ad libitum consumed feed in discrete meals lasting approximately 40 min (40.1 ± 3.9 min in summer and 41.9 ± 3.8 min in winter) (25). The shorter and longer schedules were then selected to evaluate behavioral responses to access periods below and above this reference feeding bout duration. More generally, horses in natural or semi-natural environments rarely experience prolonged fasting periods (1), while feeding bouts have been reported to range from approximately 1–3 h (2, 26). Therefore, the schedules used in the present study were intended as an exploratory assessment of how different forage access intervals may influence behavioral responses and the expression of frustration-related behaviors. Interestingly, the medium opening condition used in the present study (40 min open and 20 min closed) closely reflects the natural feeding bout duration previously reported in ponies fed a short-chopped fiber feed ad libitum, where feed was consumed as discrete meals lasting 40.1 ± 3.9 min in summer and 41.9 ± 3.8 min in winter (25). This may partly explain why the ME-OP schedule appeared to provide a more appropriate balance between forage restriction and the maintenance of natural feeding behavior. Moreover, the increase of licking and rubbing on the AFD can be a coping response when feeding motivation is high but access is limited (27), and this pattern was evident in the SH-OP condition. From a physiological perspective the progressive reduction of foraging behavior associate with the shorter access condition (SH-OP) further underscores the importance of aligning automated feeding schedules with the natural feeding motivation of horses. Given that horses are physiologically adapted to frequent, small meals throughout the day, excessively short intervals may not allow them to maintain an adequate feeding rhythm and may negatively influence their digestive physiology (28). This may also lead to an increased risk of developing stereotypic behaviors. Cooper et al. (29) showed an increase in stereotypic behaviors as meal frequencies were increased.

All these findings suggest that automated feeding systems such as the AFD may represent a promising tool for improving welfare and feeding management, provided that access intervals are sufficiently long to support natural feeding behavior and prevent stress-related behaviors. Therefore, based on the present results, a minimum continuous access time to forage of at least 40 min can be proposed when using an AFD, with a time schedule set to 40 min open and 20 min closed (ME–OP). From a practical perspective, such devices may also address management challenges previously highlighted by owners and caretakers, particularly the increased labor associated with filling and maintaining traditional slow feeders (10).

Some limitations should be considered when interpreting these results. The sample size was relatively small, particularly for the group-housed trial. Additionally, future studies should explore the use of different hay types and automated device designs to assess whether the observed effects are consistent across varying forage characteristics, group compositions, and environmental settings. Long-term studies are also needed to determine whether the behavioral patterns observed in the present study are maintained over time and to evaluate their potential effects on forage intake, body condition, health and welfare.

## 5 Conclusion

The present study demonstrated that behavioral responses to the AFD were influenced by both social dynamics and AFD access schedule. In group-housed horses, individual differences in AFD use and agonistic interactions suggested that social hierarchy played an important role in determining access to the feeding resource. These findings indicate that group composition and social stability should be considered when implementing AFDs in group-housed horses. In individually housed horses, the programmed AFD opening and closing schedule was a key determinant of behavioral responses. Shorter opening intervals reduced feeding behavior and increased stress-related behaviors directed toward the AFD, including pawing, head rubbing and oral manipulation, whereas longer opening intervals promoted more sustained foraging and lower expression of frustration-related behaviors. From the schedules tested, the medium opening interval (40 min open, 20 min closed) appeared to provide the most appropriate balance between regulating hay intake, limiting time without forage, and minimizing behavioral disturbances.

## Data Availability

The raw data supporting the conclusions of this article will be made available by the authors, without undue reservation.

## References

[1] Ellis AD. "Biological basis of behaviour and feed intake". In: Ellis AD Longland AC Coenen M Miraglia N, editors. The impact of Nutrition on the Health and Welfare of Horses. Wageningen, The Netherlands: Wageningen Academic Publishers (2010). p. 53–74.

[2] Taylor DEF Lancaster BE Ellis AD. Time budgets in domesticated male Icelandic horses on pasture turnout in winter and spring. Animals. (2025) 15:3206. doi: 10.3390/ani15213206, 41227536 PMC12610192

[3] Ellis AD Redgate S Zinchenko S Owen H Barfoot C Harris P. The effect of presenting forage in multi-layered haynets and at multiple sites on night time budgets of stabled horses. Appl Anim Behav Sci. (2015) 171:108–16. doi: 10.1016/j.applanim.2015.08.012

[4] Bordin C Raspa F Greppi M Harris P Ellis AD Roggero A . Pony feeding management: the role of morphology and hay feeding methods on intake rate, ingestive behaviors and mouth shaping. Front Vet Sci. (2024) 11:1–14. doi: 10.3389/fvets.2024.1332207, 38681853 PMC11046934

[5] Martinson K Wilson J Cleary K Lazarus W Thomas W Hathaway M. Round-bale feeder design affects hay waste and economics during horse feeding. J Anim Sci. (2012) 90:1047–55. doi: 10.2527/jas.2011-4087, 21984721

[6] Henderson AJZ. Don’t fence me in: managing psychological well being for elite performance horses. J Appl Anim Welf Sci. (2007) 10:309–29. doi: 10.1080/10888700701555576, 17970632

[7] Glunk EC Hathaway MR Grev AM Lamprecht ED Maher MC Martinson KL. The effect of a limit-fed diet and slow-feed hay nets on morphometric measurements and postprandial metabolite and hormone patterns in adult horses. J Anim Sci. (2015) 93:4144–52. doi: 10.2527/jas.2015-9150, 26440194

[8] Luthersson N Hou Nielsen K Harris P Parkin TDH. Risk factors associated with equine gastric ulceration syndrome (EGUS) in 201 horses in Denmark. Equine Vet J. (2009) 41:625–30. doi: 10.2746/042516409X441929, 19927579

[9] Hothersall B Nicol C. Role of diet and feeding in Normal and stereotypic behaviors in horses. Vet Clin North Am Equine Pract. (2009) 25:167–81. doi: 10.1016/j.cveq.2009.01.002, 19303558

[10] Roig-Pons M Bachmann I Freymond SB. Impact of feeding strategies on the welfare and behaviour of horses in groups: an experimental study. PLoS One. (2025) 20:e0325928. doi: 10.1371/journal.pone.0325928, 40560838 PMC12193674

[11] Seabra JC Hess T Martinez do Vale M Spercoski KM Brooks R Dittrich JR. Effects of different hay feeders, availability of roughage on abnormal behaviors and cortisol circadian rhythm in horses kept in dry lots. J Equine Vet Sci. (2023) 130:104911. doi: 10.1016/j.jevs.2023.104911, 37625626

[12] Hampson BA De Laat MA Monot J Bailliu D Pollitt CC. Adaption of horses to a novel dynamic feeding system: movement and behavioural responses. Equine Vet J. (2013) 45:481–4. doi: 10.1111/evj.12002, 23216599

[13] Kjellberg L Morgan K. Introduction to automatic forage stations and measurement of forage intake rate in an active open barn for horses. Animal. (2021) 15:100152. doi: 10.1016/j.animal.2020.100152, 33573955

[14] Mazzola S Palestrini C Cannas S Fè E Bagnato GL Vigo D . Efficacy of a feed dispenser for horses in decreasing cribbing behaviour. Vet Med Int. (2016) 2016:1–6. doi: 10.1155/2016/4698602, 27818832 PMC5081461

[15] Bass L Swain E Santos H Hess T Black J. Effects of feeding frequency using a commercial automated feeding device on gastric ulceration in exercised quarter horses. J Equine Vet Sci. (2018) 64:96–100. doi: 10.1016/j.jevs.2018.02.005, 30973160

[16] Bordin C Raspa F Harris P Ellis AD Roggero A Palestrini C . Effect of pony morphology and hay feeding methods on back and neck postures. J Anim Physiol Anim Nutr. (2023) 180:3–14. doi: 10.1111/jpn.13861 37452526

[17] Valle E Raspa F Giribaldi M Barbero R Bergagna S Antoniazzi S . A functional approach to the body condition assessment of lactating donkeys as a tool for welfare evaluation. PeerJ. (2017) 5:e3001. doi: 10.7717/peerj.3001, 28367363 PMC5372837

[18] Henneke DR Potter GD Kreider JL Yeates BF. Relationship between condition score, physical measurements and body fat percentage in mares. Equine Vet J. (1983) 15:371–2. doi: 10.1111/j.2042-3306.1983.tb01826.x, 6641685

[19] Harris PA Ellis AD Fradinho MJ Jansson A Julliand V Luthersson N . Review: feeding conserved forage to horses: recent advances and recommendations. Animal. (2017) 11:958–67. doi: 10.1017/S1751731116002469, 27881201

[20] Friard O Gamba M. BORIS: a free, versatile open-source event-logging software for video/audio coding and live observations. Methods Ecol Evol. (2016) 7:1325–30. doi: 10.1111/2041-210X.12584

[21] Baumgartner M Erhard MH Zeitler-Feicht MH. Which animal-to-feeding-place ratio at time-controlled hay racks is animal appropriate? Preliminary analysis of stress responses of horses. Front Vet Sci. (2023) 9:1005102. doi: 10.3389/fvets.2022.1005102, 36699339 PMC9868146

[22] Jørgensen GHM Borsheim L Mejdell CM Søndergaard E Bøe KE. Grouping horses according to gender-effects on aggression, spacing and injuries. Appl Anim Behav Sci. (2009) 120:94–9. doi: 10.1016/j.applanim.2009.05.005

[23] Mata F Boyton G Young T Mata F Boyton G Young T. Anticipatory behaviour during the approach to feeding times as a measure of horse welfare. Animals. (2024) 14:14. doi: 10.3390/ani14243677 PMC11672559 39765580

[24] Ince J Longland AC Newbold JC Harris PA. Changes in proportions of dry matter intakes by ponies with access to pasture and haylage for 3 and 20 hours per day respectively, for six weeks. J Equine Vet Sci. (2011) 31:283. doi: 10.1016/j.jevs.2011.03.106

[25] Dugdale AHA Curtis GC Cripps PJ Harris PA Argo CMG. Effects of season and body condition on appetite, body mass and body composition in ad libitum fed pony mares. Vet J. (2011) 190:329–37. doi: 10.1016/j.tvjl.2010.11.009, 21146430

[26] Benhajali H Richard-Yris M-A Ezzaouia M Charfi F Hausberger M. Foraging opportunity: a crucial criterion for horse welfare? Animal. (2009) 3:1308–12. doi: 10.1017/S1751731109004820, 22444907

[27] Rochais C Henry S Hausberger M. “Hay-bags” and “slow feeders”: testing their impact on horse behaviour and welfare. Appl Anim Behav Sci. (2018) 198:52–9. doi: 10.1016/j.applanim.2017.09.019

[28] Nadeau JA Andrews F Patton CS Argenzio RA Mathew AG Saxton AM. Effects of hvdrochloric, valeric, and other volatile fatty acids on pathogenesis of ulcers in the nonglandular portion of the stomach of horses. Am J Vet Res. (2003) 64:413–7. doi: 10.2460/ajvr.2003.64.413, 12693529

[29] Cooper JJ Mcall N Johnson S Davidson HPB. The short-term effects of increasing meal frequency on stereotypic behaviour of stabled horses. Appl Anim Behav Sci. (2005) 90:351–64. doi: 10.1016/j.applanim.2004.08.005

